# Fermentable carbohydrates intake and dental caries in adolescents under household food insecurity: a counterfactual analysis

**DOI:** 10.1007/s40368-026-01195-x

**Published:** 2026-03-19

**Authors:** D. L. da Silveira, J. K. Knorst, B. Brondani, É. Silva Rios, L. N. Guidolin, F. C. Fraiz, T. M. Ardenghi

**Affiliations:** 1 Federal University of Santa Maria, Santa Maria, Brazil; 2 Federal University of Paraná, Curitiba, Brazil

**Keywords:** Dental caries, Food insecurity, Oral health, Social inequalities

## Abstract

**Purpose:**

To evaluate the association between fermentable carbohydrate consumption and occurrence of dental caries according to the level of food insecurity (FI) at home.

**Methods:**

Cross-sectional study nested within a population-based cohort that began in 2010 with Brazilian preschoolers. Data were from the fifth follow-up wave, including adolescents aged 14–18 years. Dental caries was assessed using the International Caries Detection and Assessment System (ICDAS), considering the number of cavitated teeth. FI was measured with the Brazilian Food Insecurity Scale (EBIA). Fermentable carbohydrate consumption was categorized by self-reported frequency of cariogenic food intake. Demographic and socioeconomic variables were collected as confounders. Associations were estimated using Poisson regression, overall and stratified by FI status, and presented as Mean Ratios (MR) with 95% confidence intervals (CI). A non-linear Blinder-Oaxaca decomposition was applied to explore the FI impact.

**Results:**

The sample comprised 300 adolescents. After adjustment, daily fermentable carbohydrates consumption was associated with more cavitated lesions (MR 2.02; 95% CI 1.46–2.81). In the stratified analysis, the association was stronger among those with FI (MR 4.88; 95% CI 1.99–11.96), but not significant in adolescents without FI (MR 1.18; 95% CI 0.75–1.86). The decomposition showed a 95% difference in caries levels between fermentable carbohydrates intake groups; 16% was explained by observed variables mainly household income (9.5%) and FI (6.5%) and 72% by unmeasured factors.

**Conclusion:**

Daily fermentable carbohydrates consumption in FI adolescents is associated with higher rates of cavitated carious lesions, reinforcing the need for policies integrating dietary habits and food security to improve oral health outcomes.

**Supplementary Information:**

The online version contains supplementary material available at 10.1007/s40368-026-01195-x.

## Introduction

Despite being preventable, dental caries remains one of the most common chronic diseases globally, with a particularly high burden among children and adolescents, and significant functional, social, and economic consequences (Splieth et al. 2020; Costa et al. 2024; Peres et al. 2019). Among its main risk factors, the frequent and excessive consumption of fermentable carbohydrates stands out, which plays a central role in favoring the imbalance of dental biofilm and the demineralization of dental tissues (Costa et al. 2024). However, the effects of fermentable carbohydrates consumption on caries do not occur in isolation, but are modulated by unfavorable socioeconomic conditions, such as food insecurity (FI), low education, and limited access to preventive care. Thus, these social determinants of health create conditions that increase exposure to risk and compromise effective disease control (Bezerra et al. 2024; Feldens et al. 2025).

The intensification of oral health problems in the context of FI can result from multiple interconnected factors. Limited or uncertain access to food often leads to a diet low in non-cariogenic foods, such as fruits, vegetables, and dairy, and higher consumption of cheap, sugar-rich items. In addition, populations experiencing FI may have reduced access to oral health care, including preventive dental check-ups. The combination of these factors can exacerbate the deleterious effects of fermentable carbohydrate intake on oral health, particularly among vulnerable groups (Sabbagh et al. 2023). Growing evidence has documented consistent associations between FI and elevated dental caries prevalence in children, adolescents, and adults across different populations and contexts (Holmes 2024; Bahanan et al. 2021; Santin et al. 2016; Ferreira et al. 2019; Drumond et al. 2023), suggesting that FI may act as an independent risk factor for oral health beyond the effects of low income alone.

According to the 2023 Continuous National Household Sample Survey (Continuous PNAD), conducted by the Brazilian Institute of Geography and Statistics (IBGE), 27.6% of Brazilian households—about 21.6 million—experienced some degree of FI, affecting more than 64 million people (IBGE 2024). Severe FI, characterized by hunger and disrupted eating patterns, reached 4.1% of households (approximately 3.2 million), impacting both adults and children. FI contributes to malnutrition, cognitive deficits, increased susceptibility to infections, and impaired development, perpetuating cycles of poverty and overburdening health and social support systems (IBGE 2024). Previous results have shown that individuals with FI tend to consume ultra-processed beverages and foods more frequently, which are more economically accessible and rich in fermentable carbohydrates, while facing greater barriers to dental care and the adoption of preventive practices (da Silva et al. 2023). In this context, these ready-to-consume, non-perishable, low-cost, highly palatable, and easily marketable ultra-processed foods that are high in fermentable carbohydrates has been increasing among these populations (Leung et al. 2022). Their characteristics make them convenient and competitive with fresh foods, contributing to their high consumption among the most vulnerable groups (Monteiro et al. 2019).

Despite the biological and social plausibility of this interaction, evidence evaluating the influence of high fermentable carbohydrates consumption on dental caries considering the level of food insecurity is still scarce, which highlights the need for studies that incorporate these social determinants into the understanding of the oral health-disease process (Sabbagh et al. 2023). This study aimed to assess the association between fermentable carbohydrates consumption and dental caries, with a specific focus on how this relationship varies according to household food insecurity. We hypothesized that adolescents with daily fermentable carbohydrates intake living in food-insecure households would exhibit higher dental caries levels compared to those with similar consumption but no FI exposure.

## Methods

### Reporting and ethics procedures

This study followed the guidelines of STROBE (Strengthening the Reporting of Observational Studies in Epidemiology) and was approved by the Research Ethics Committee of the Federal University of Santa Maria (CAAE: 63937422.0.0000.5346; Opinion No. 5,698,152), in accordance with Resolution No. 466/2012 of the National Health Council. All participants or their legal guardians, signed the Informed Consent Form.

### Study design and population

This is a cross-sectional study conducted in a population-based cohort of preschool children living in the municipality of Santa Maria, southern Brazil. The municipality of Santa Maria is considered a medium-sized city, with a fluoridated water supply and a significant influence in the central region of the state, being the 54th highest-income city in the country (Brondani et al. 2025). The initial assessment of the cohort was conducted in 2010 with children aged 1–5 years without any cognitive disorder or disability, who were selected through systematic probability sampling in 15 primary health care units equally distributed across the eight administrative regions of the city. Subsequently, four subsequent assessments in 2012, 2017, 2020, and 2023 were performed, and the present study used data from the last wave of the cohort (2023), when participants were between 14 and 18 years old. Further details on the cohort study design and procedures can be found in previously published studies (Guedes et al. 2016; Knorst et al. 2022; Pohl et al. 2024).

The sample size calculation for the baseline study has been published previously (Piovesan et al. 2012). A post hoc power analysis was conducted considering the observed Mean Ratio (MR = 4.88) for the association between daily fermentable carbohydrates consumption and the number of cavitated carious lesions among adolescents experiencing FI. The analysis included approximately 23 participants in the rare-consumption group and 145 in the daily-consumption group. Assuming a significance level of α = 0.05, the statistical power was estimated to be greater than 95%.

### Data collection and research team calibration

Data collection for this study was carried out throughout 2023. During this period, the adolescents were examined in their schools or at their homes. The contact information used to locate the participants was obtained in the previous phases of the cohort and included data from the municipality’s enrollment center, contact with parents or guardians through telephone calls, social networks, and home visits. The training and calibration for dental caries followed the standardized methodology guidelines for epidemiological surveys, involving theoretical and practical classes, as well as specific calibration sessions (World Health Organization 2013). Examiner agreement was assessed using the Kappa statistic, with values ranging from 0.70 to 0.81 for inter-examiner and from 0.70 to 0.88 for intra-examiner (Ismail et al. 2007).

### Dental caries assessment

The presence of dental caries lesions was evaluated through clinical examinations, using the diagnostic criteria of the *International Caries Detection and Assessment System* (ICDAS) (Ismail et al. 2007). Initially, the tooth surfaces were examined wet and then dried with gauze, using a CPI probe (or ballpoint pen) and dental mirror, under natural lighting. When plaque accumulated, prophylaxis was performed with a toothbrush. For the purpose of analysis, only surfaces with cavitated caries lesions, corresponding to ICDAS scores 3, 5, and 6, were considered, both in terms of the total number of affected surfaces and the prevalence of these lesions in the sample. This restriction is justified because these scores correspond to cavitated lesions or those with potential for clinical intervention, i.e., lesions requiring direct therapeutic attention. Previous studies have also adopted this approach to focus on lesions of greater clinical relevance, allowing for a more precise assessment of the impact of risk factors on dental caries (Brondani et al. 2023, 2024, 2025).

### Fermentable carbohydrate intake

The frequency of consumption of cariogenic ultra-processed foods, which are industrial formulations high in sugars, fats, and additives with little or no whole foods, was assessed through face-to-face interviews with the adolescents using a questionnaire listing common items and their frequency of consumption, with fermentable carbohydrate intake referring to foods high in free or added sugars. The list of food items was based on the report published by the Pan American Health Organization (PAHO) on ultra-processed foods consumed in Latin America (Pan American Health Organization 2019). We included only the five ultra-processed foods identified as the main sources of free sugars in the PAHO report (Pan American Health Organization 2019): soft drinks or chocolate drinks, sweets (candies, caramels, lollipops, ice cream, chocolate), cakes, cookies/filled cookies, and potato chips. While other fermentable carbohydrates can also contribute to caries, we focused on the foods that contribute most to free sugar intake in the population.

The frequency of consumption of ultra-processed foods high in fermentable carbohydrates, specifically sweets, soda, cake, cookies, and chocolate milk, were recorded as 0 = almost never or rarely, 1 = sometimes during the week, and 2 = every day or almost every day (Crema et al. 2023). Consumption was categorized into two groups, based on the scores obtained from the self-reported frequency. Participants who reported “never,” “rarely,” or “sometimes” consumption were classified as having rare consumption. Those who reported “every day” or “almost every day” consumption were classified as having daily consumption. It is important to clarify that our approach assessed consumption frequency of these specific foods rather than direct measurement of total sugar or fermentable carbohydrate intake through quantitative dietary assessment methods. Oral hygiene was assessed according to toothbrushing in the last 30 days: not brushing daily, once a day, twice a day, three times a day, or four or more times a day.

### Assessment of food insecurity

The assessment of FI at home was carried out using the Brazilian Food Insecurity Scale (*EBIA*) (Ismail et al. 2007). This tool investigates experiences related to the lack of food in the residence in the last three months, ranging from apprehension about the possibility of lack of food to the situation of spending an entire day without food (Segall-Corrêa et al. 2014). The EBIA, answered based on the report of the head of household, consists of 14 yes/no questions, aimed at families with residents under 18 years of age, with one point being assigned for each affirmative answer, totaling a score between 0 and 14 (Pérez-Escamilla et al. 2004). Based on the score obtained, households are classified into four categories: food security (0 points), mild food insecurity (1 to 5 points), moderate (6 to 9 points) and severe (10 to 14 points) food insecurity. For the regression analyses, the food security categories were grouped into two groups: households in a situation of food security (0—without FI) and households with some degree of food insecurity (mild, moderate or severe—with FI) (Segall-Corrêa et al. 2014).

### Covariates of interest

Demographic and socioeconomic characteristics were collected by self-reported questionnaires. For this, sex (female or male) and age (in years) were evaluated. Additionally, skin color was assessed according to the Brazilian Institute of Geography and Statistics (IBGE) criteria and categorized as white and brown-black-yellow. Household income was collected in Brazilian *reais*—R$, considering the sum of all family earnings in the month and categorized according to the Brazilian minimum wage (BMW)—adolescents from families earning less than 1 BMW or more than 1 BMW. The value of the BMW is R$ 1412.00, and US$1.00 is equivalent to R$5.52. Dental attendance was dichotomized into: routine care (comprising preventive examinations or orthodontic treatment) and non-routine care (prompted by toothache, other orofacial pain, accidents, or trauma).

### Data analysis

Data were analyzed using Stata version 18. Descriptive statistics were used to summarize demographic, socioeconomic, behavioral, and clinical characteristics of the participants. A comparative analysis was performed between the participants evaluated and those lost at last follow-up. The association between fermentable carbohydrates consumption and the number of cavitated carious lesions was assessed using Poisson regression models. Analyses were conducted for the general sample and stratified by FI status. Covariates were selected a priori based on their theoretical relevance and guided by the conceptual framework proposed by the Commission on Social Determinants of Health (Solar and Irwin 2010). Variables with p < 0.20 were eligible for the adjusted model, and those that were significant were maintained in the final parsimonious model. Results were presented as a Mean Ratio (MR) and 95% confidence interval (95% CI).

To explore disparities in dental caries experience, a counterfactual decomposition analysis was conducted using the non-linear Blinder-Oaxaca method (Sinning et al. 2008). This method decomposes the observed difference in the mean number of cavitated carious lesions between adolescents with rare and daily frequency of fermentable carbohydrates consumption into two components: an explained component (attributable to differences in observable characteristics such as household income and FI status) and an unexplained component (attributable to differences in coefficients or unmeasured factors). The group with rare fermentable carbohydrates consumption was used as the reference. Both absolute and relative contributions of explanatory variables to the total difference were reported.

## Results

A total of 300 adolescents were evaluated (about 50% of original sample). The comparative analysis between participants who completed the study and those who dropped out did not reveal statistically significant differences in the main variables of interest (p > 0.05), suggesting that loss to follow-up did not compromise the study results (Supplementary Table 1). Most participants were female (55.0%), with the mean age of 15.7 (SD 1.3) year-olds. The majority self-identified as White (77.1%), while the remaining reported Black, Brown, or Yellow skin color (22.9%). Most adolescents resided in households with a monthly income exceeding one Brazilian minimum wage (BMW) (73.8%) and reported regular dental visits (62.7%). Regarding dietary habits, 84.9% of the sample reported daily consumption of foods that are high in fermentable carbohydrates. Food Insecurity was present in 66.0% of households. The mean number of surfaces with cavitated carious lesions was 1.45 (Standard Deviation [SD] = 3.58) (Table 1).

**Table 1:** General sample characteristics of the sample, Santa Maria, Brazil, N = 300

Variables	N^a^	%
Sex		
Female	165	55.0
Male	135	45.0
Age (years)		
14	75	18.9
15	83	20.9
16	91	22.9
17	148	37.3
Skin color		
White	306	77.1
Black-brown-yellow	91	22.9
Household income (in *Reais*)		
≥ 1 BMW	220	73.8
< 1 BMW	78	26.2
Dental attendance		
Regular	254	62.7
Non-regular	151	37.3
Frequency of fermentable carbohydrate consumption		
Rare	61	15.1
Daily	344	84.9
Food security status		
Present	132	44.0
Absent	168	66.0
Outcome	Mean	SD
Number of surfaces with cavitated lesions	1.45	3.58

*SD* standard deviation

^a^Values lower than 406 are due to missing data

Table 2 presents the mean number of cavitated carious lesions according to fermentable carbohydrates consumption and FI status. In the general sample, adolescents who consumed fermentable carbohydrates daily had a higher mean number of cavitated lesions (1.52; SD 3.75) than those with rare consumption (1.09; SD 2.48). Among adolescents without FI, the mean number of lesions was similar regardless of fermentable carbohydrates consumption (0.88 vs. 1.00). However, among those with FI, adolescents with daily fermentable carbohydrates intake had a markedly greater number of lesions (2.27; SD 5.07) compared to those with rare intake (0.65; SD 1.11).

**Table 2:** Mean of surfaces with cavitated lesions in adolescents according to frequency of fermentable carbohydrate consumption and presence of food insecurity, Santa Maria, N = 300

Frequency of fermentable carbohydrate consumption	Surfaces with cavitated caries lesions Mean (SD)
	General sample (N = 300)
Rare	1.09 (2.48)
Daily	1.52 (3.75)
	Adolescents without FI (n = 132)
Rare	0.88 (1.67)
Daily	1.00 (2.46)
	Adolescents with FI (n = 168)
Rare	0.65 (1.11)
Daily	2.27 (5.07)

*SD* standard deviation, *FI* food insecurity

Table 3 presents the association between frequency of fermentable carbohydrates consumption and the number of surfaces with cavitated carious lesions. In the general sample (Model 1), adolescents who consumed fermentable carbohydrates daily had a higher number of cavitated lesions compared to those with rare fermentable carbohydrates intake, both in the crude model (MR 1.38; 95%CI: 1.07–1.78) and after adjustment for sex, age, skin color, and household income (MR 2.02; 95%CI: 1.46–2.81). Among adolescents without FI (Model 2), no significant association was observed between fermentable carbohydrates consumption and cavitated lesions, either in the crude (MR 1.12; 95%CI: 0.72–1.75) or adjusted model (MR 1.18; 95%CI: 0.75–1.86). Conversely, among adolescents experiencing FI (Model 3), daily fermentable carbohydrates consumption was strongly associated with a higher number of cavitated lesions. The crude model showed a mean ratio of 3.48 (95%CI: 2.07–5.84), and this association remained significant after adjustment for sex, age, household income, and dental attendance (MR 4.88; 95%CI: 1.99–11.96).

**Table 3:** Association between fermentable carbohydrate consumption and cavitated caries lesions in adolescents in general sample and stratified according to food security status

Frequency of fermentable carbohydrate consumption	Number of surfaces with cavitated caries lesions			
	Crude MR (95%CI)	p-value	Adjusted MR (95%CI)	p-value
	Model 1	General sample (N = 300)		
Rare	1 (reference)		1 (reference)	
Daily	1.38 (1.07–1.78)	< 0.05	2.02 (1.46–2.81)	< 0.01
	Model 2	Adolescents without FI (n = 132)		
Rare	1 (reference)		1 (reference)	
Daily	1.12 (0.72–1.75)	0.603	1.18 (0.75–1.86)	0.480
	Model 3	Adolescents with FI (n = 168)		
Rare	1 (reference)		1 (reference)	
Daily	3.48 (2.07–5.84)	< 0.01	4.88 (1.99–11.96)	< 0.01

*MR* mean ratio, *CI* confidence interval, *FI* food insecurity

Model 1: adjusted for sex, age, skin color and household income; Model 2: adjusted for sex, age, skin color and household income; Model 3: adjusted for sex, age, household income and dental attendance

Table 4 presents the decomposition of the effect of fermentable carbohydrates consumption on the mean number of cavitated carious lesions, based on the Blinder-Oaxaca model. The model explains a 95% difference in dental caries levels between fermentable carbohydrates intake groups. Of this gap, 16% (−0.16/−0.95) was explained by the observed variables included in the model, while the remaining 72% (−0.78/−0.95) was attributed to unobserved or unmeasured factors. This indicates that if adolescents with daily fermentable carbohydrates consumption had the same household income and FI status as those with rare fermentable carbohydrates consumption, the gap would be reduced by 16%. Among the explained components, household income accounted for 9.5% of the difference, and food insecurity status explained 6.5%. Although we adjusted for key sociodemographic variables and dental attendance, future studies that consider broader dietary patterns, such as portion size, consumption of other fermentable foods and sugary or acidic beverages, intake between meals, protective dietary components, and oral hygiene practices including fluoride exposure could provide a more comprehensive understanding of the determinants of dental caries in adolescents.

**Table 4:** Effect decomposition analysis according to fermentable carbohydrate consumption and dental caries determined by Blinder-Oaxaca model

Mean of surfaces with cavitied caries lesions	Coefficient (IC 95%)^a^	Percentage
Dental caries in rare fermentable carbohydrate frequency group	0.78 (0.38; 1.17)	
Dental caries in daily fermentable carbohydrate frequency group	1.73 (1.20; 2.26)	
Difference	−0.95 (−1.61; −0.29)	
Difference due to model variables	−0.16 (−0.40; 0.07))	16.0%
Differences not explained by the model	−0.78 (−1.40; −0.17)	72.0%
Detailed analysis of the part explained by the model		
Household income	−0.09 (−0.29; 0.09)	9.5%
Food insecurity status	−0.06 (−0.19; 0.05)	6.5%

*IC* confidence interval

^a^Mean of surfaces with cavitied caries lesions estimated by the Blinder-Oaxaca pooled model

## Discussion

This study evaluated the association between fermentable carbohydrates consumption and dental caries levels, with emphasis on the differences according to the level of FI at home. Our findings are in agreement with the conceptual hypothesis of the study, showed that adolescents with daily fermentable carbohydrates consumption and exposed to FI situations presented a higher number of cavitated caries lesions, compared to those with the same consumption pattern, but living in food security. Our findings also demonstrate that differences between consumption profile and dental caries can be partially explained by FI and family income. These findings reinforce the importance of considering social determinants in understanding the etiology of dental caries and indicate that FI can potentiate the harmful effects of excessive fermentable carbohydrates consumption on oral health.

Adolescents who consumed fermentable carbohydrates daily had a higher number of cavitated lesions compared to those with rare fermentable carbohydrates intake. The relationship between fermentable carbohydrates consumption and dental caries is well established and dose-dependent, i.e., the greater the frequency and amount of fermentable carbohydrates intake, the greater the risk of developing caries lesions (Tinanoff et al. 2019; Feldens et al. 2025). In particular, sucrose consumption is widely recognized as one of the main etiological factors of the disease (Sheiham and James 2014; WHO 2015). This disaccharide acts as a preferred substrate for cariogenic microorganisms, which metabolize it generating organic acids capable of reducing the pH of dental plaque to critical levels, promoting enamel demineralization (Peres et al. 2016). In addition, the frequency and pattern of fermentable carbohydrates consumption directly influence the de-remineralization process, favoring the progression of lesions (Machiulskiene et al. 2020). Although the consumption of fermentable carbohydrates is strongly associated with the development of dental caries, it is important to note that this is not the only determining factor of the condition. Evidence from previous research indicates that socioeconomic and behavioral factors can modify or enhance the effect of fermentable carbohydrates consumption on dental caries (Tinanoff et al. 2019; Peres et al. 2016; Feldens et al. 2025).

Our findings suggest that differences in dental caries experience according to consumption of ultra-processed foods high in fermentable carbohydrates, classified as rare versus daily frequency, can be partly explained by food insecurity and family income. In our sample, daily consumption of these specific ultra-processed foods was associated with greater caries experience, particularly among individuals with lower income and those experiencing FI. These results emphasize the influence of social determinants on the etiology of dental caries and suggest that FI may exacerbate the harmful effects of frequent fermentable carbohydrate consumption from these foods, leading to substantially higher caries levels.

Also, this association may be explained by the fact that food insecurity is related to increased intake of fermentable carbohydrates (Holmes 2024; Ferreira et al. 2019; Santin et al. 2016) and, consequently, a higher occurrence of dental caries. Among food-insecure populations, the consumption of ultra-processed foods rich in fermentable carbohydrates is more frequent due to their lower cost and wider availability (Bahanan et al. 2021; Romão et al. 2025). In addition, FI is commonly associated with barriers to accessing oral health services, less frequent preventive practices such as toothbrushing and fluoride use, and greater overall social vulnerability (Bezerra et al. 2020; Gois et al. 2024). As a result, similar levels of fermentable carbohydrate intake may have more detrimental effects on the oral health of food-insecure individuals than on those who are food-secure (Holmes 2024; Sabbagh et al. 2023).

However, the estimated fourfold increase in caries among adolescents experiencing both FI and daily fermentable carbohydrate consumption should be interpreted with caution. This estimate may be unstable and possibly overestimated due to the small number of food-insecure adolescents with rare fermentable carbohydrate intake in our sample, leading to wide confidence intervals and greater uncertainty around the effect size.

On the other hand, adolescents with daily fermentable carbohydrates intake but without context of FI at home did not present a significant higher occurrence of dental caries. This result suggests that food security may attenuate the effect of exposure on the outcome, possibly because it is associated with a set of protective factors. Families without FI tend to have greater access to health information, regular dental care, healthier eating practices, higher family income, and better oral hygiene habits (Jackson and Testa 2021), which contributes to reducing susceptibility to the disease even in the face of high exposure to fermentable carbohydrates (Camargo et al. 2012; Ardenghi et al. 2012).

The decomposition analysis indicated that household income and FI explained a portion of the variation in dental caries levels according to fermentable carbohydrates intake level. Household income, in addition to representing an indicator of socioeconomic status, directly reflects the ability to access essential goods and services (Camargo et al. 2012; Ardenghi et al. 2012; Zajacova and Lawrence 2018; Alwadi and Vettore 2019). It expresses structural inequalities that influence the living and health conditions of families, being associated with greater social vulnerability and worse health indicators (Camargo et al. 2012; Ardenghi et al. 2012). Thus, people from low socioeconomic background have a higher likelihood of non-preventive dental visits (Zajacova and Lawrence 2018; Alwadi and Vettore 2019; Gurutana et al. 2014), less access to healthy foods, in addition to having a higher consumption of foods ultra-processed and high in fermentable carbohydrates (Valenzuela et al. 2021). The situation worsens when it is associated with FI, which food choices are strongly conditioned by financial availability and the need to prioritize foods with high energy density and low cost, generally poor in nutrients and rich in fermentable carbohydrates (Drumond et al. 2023), impacting dental caries levels (Sabbagh et al. 2023; Holmes 2024; Bahanan, et al. 2021).

This study has some limitations that should be considered in the interpretation of the results. First, the FI assessment was carried out using self-reported measures, based on the report of the head of household, which may have resulted in underreporting of the actual condition due to embarrassment, denial or lack of direct observation. This limitation arises because the head of the household does not always observe all situations related to family members’ eating habits, as adolescents may have meals at school or outside the home, making it difficult to obtain a complete understanding of the household’s overall food conditions. However, it was assessed using a widely used and validated questionnaire for this purpose. In addition, because the EBIA questionnaire assesses food insecurity only over the past three months, it may not capture chronic patterns that more strongly affect dental caries development, potentially underestimating the association between persistent food insecurity and oral health outcomes.

Regarding diet, the estimation of fermentable carbohydrate intake was also obtained through self-report. This approach, although useful for indicating overall consumption patterns, does not allow a comprehensive assessment of the nutritional quality of the diet, as it is limited to a specific aspect of dietary behavior and does not capture the frequency or timing of consumption throughout the day (e.g., between meals or within mealtimes), nor whether oral care was performed after eating. The use of a fermentable carbohydrate intake frequency scale also presents limitations. Due to the nature of data collection, it was not possible to identify individuals with intakes of multiple fermentable carbohydrates per day or to account for portion size. This may have reduced the sensitivity to detect differences in dietary patterns across levels of food security. Furthermore, the scale does not capture potential changes in fermentable carbohydrate consumption throughout childhood, which may limit the understanding of how food insecurity influences trajectories of food intake. Future research should use others instruments to assess portion sizes and consumption patterns across different food insecurity contexts. Longitudinal studies with repeated measures, validated dietary assessments, and larger sample sizes are needed to better clarify the temporal relationship between fermentable carbohydrate intake, food insecurity, and dental caries. Finally, since this study is nested within a cohort, follow-up losses may compromise the representativeness of the original sample. However, sensitivity and power analyses confirm the validity of our findings.

Despite these limitations, the study makes a relevant contribution to investigating the effects of fermentable carbohydrate intake, FI and dental caries during adolescence, contributing to the literature on social determinants and inequalities in oral health. By highlighting how the effects of fermentable carbohydrates consumption on caries are amplified in FI contexts, the findings emphasize the importance of intersectoral actions that integrate oral health and food security. In addition, it stands out for its methodological rigor, with the use of population data, validated instruments and adequate statistical analysis, ensuring robustness to the results presented. Future longitudinal studies are encouraged to explore the causality of relationships, as well as the plausibility of possible interventions and policies in this context.

In conclusion, fermentable carbohydrates consumption was associated with caries levels, but this relationship was modified by food security status. Adolescents with daily fermentable carbohydrates intake and exposed to FI had significantly more cavitated carious lesions than their peers with similar consumption but living in food-secure conditions. Household income may interact with food security and partially explain these disparities. The findings indicate that food security can be considered a sensitive indicator of social inequities, in addition to reinforcing its strategic role in public health policies aimed at equity and the promotion of oral health.

## Supplementary Information

Below is the link to the electronic supplementary material.

Supplementary file1 (DOCX 16 KB)

## Data Availability

The data supporting this study’s findings are not publicly available due to their containing information that could compromise the privacy of research participants but are available from the corresponding author upon reasonable request.
