# Supplementary material for: Fermentable carbohydrates intake and dental caries in adolescents under household food insecurity: a counterfactual analysis
Source: Eur Arch Paediatr Dent. 2026 Mar 19;27(4):1171–9. doi: 10.1007/s40368-026-01195-x (PMC13461862; doi:10.1007/s40368-026-01195-x)
Supplement: Supplementary file 1 [file 40368_2026_1195_MOESM1_ESM.docx]

**Supplementary Table 1.** Comparison of main baseline characteristics of adolescents followed and dropouts in study

|  | **Followed** | **Dropouts** |  |
| --- | --- | --- | --- |
| **Variables** | **N (%)** | **N (%)** | **p-value*** |
|  |  |  |  |
| Sex |  |  | 0.051 |
| Female | 134 (49.8) | 135 (50.1) |  |
| Male | 117 (41.5) | 165 (58.5) |  |
| Age |  |  | 0.227 |
| 1 | 53 (47.3) | 59 (52.7) |  |
| 2 | 48 (44.4) | 60 (55.6) |  |
| 3 | 72 (51.4) | 68 (48.6) |  |
| 4 | 78 (40.8) | 113 (59.1) |  |
| Skin color |  |  | 0.297 |
| White | 204 (46.6) | 234 (53.4) |  |
| Black-brown-yellow | 46 (41.1) | 66 (58.9) |  |
| Household income (in *Reais*) |  |  | 0.637 |
| ≥ 1 BMW | 86 (46.7) | 98 (53.2) |  |
| < 1 BMW | 148 (44.5) | 184 (55.4) |  |
| Prevalence of cavitated dental caries |  |  | 0.748 |
| No | 159 (45.0) | 194 (55.0) |  |
| Yes | 92 (46.5) | 106 (54.5) |  |

BMW, Brazilian minimum wage – equivalent to 1,412 *reais*; *Chi-square test.
